# Treatment of Vancouver B2 Femur Fractures With Open Reduction Internal Fixation Versus Revision Arthroplasty

**DOI:** 10.7759/cureus.38614

**Published:** 2023-05-05

**Authors:** Michael Perry, John-Luke Rivera, Michael Wesolowski, Carlo Eikani, William Lack, Joseph Cohen, Nicholas Brown

**Affiliations:** 1 Department of Orthopedic Surgery, Scripps Green Hospital, La Jolla, USA; 2 Department of Orthopedic Surgery and Rehabilitation, Loyola University Medical Center, Maywood, USA; 3 Department of Orthopedic Surgery, University of Washington, Seattle, USA

**Keywords:** open reduction with internal fixation, total hip arthroplasty: tha, revision arthroplasty, periprosthetic femur fracture, vancouver type b2

## Abstract

Background

Vancouver B2 periprosthetic femur fractures have traditionally been treated with revision arthroplasty. However, there is increasing evidence that open reduction and internal fixation (ORIF) may be a valid alternative treatment strategy. The purpose of this study was to compare the outcomes of ORIF versus revision arthroplasty for the treatment of Vancouver B2 fractures and evaluate the influence of the treating surgeon’s fellowship training on treatment selection.

Methodology

This was a retrospective cohort study of 31 patients treated for Vancouver B2 periprosthetic fractures (16 ORIF and 15 revision arthroplasty) at a single academic Level 1 trauma center. Outcome measures included one-year mortality, revision, reoperation, infection, and blood loss.

Results

There were no statistically significant differences in revision, reoperation, or infection at an average follow-up of 65 weeks. Median estimated blood loss was higher in the arthroplasty group (700 cc versus 400 cc; *P* = 0.04). There were five deaths in the ORIF group versus one in the revision group (*P *= 0.18). Cases treated by surgeons with fellowship training in arthroplasty were more likely to be treated with revision arthroplasty (10/11, 90.9%) than those treated by surgeons with fellowship training in trauma (5/15, 33.3%; *P *< 0.01).

Conclusions

There was no difference in outcomes between the two treatment strategies, but revision was associated with higher blood loss. The appropriate treatment method should be based on surgeon familiarity and patients' characteristics.

## Introduction

The incidence of periprosthetic femur fractures continues to increase with the growing volume of total hip arthroplasty (THA) [1-3]. The incidence approaches 5% at 15 to 20 years after primary THA [4]. These fractures are associated with mortality rates similar to that of traditional hip fractures but with higher short-term complications and reoperation rates [5-6].

The Vancouver classification for postoperative periprosthetic femur fractures is a validated method of classification, which has traditionally guided the treatment of these fractures [4]. Periprosthetic fractures that occur around the stem of the femoral component are classified as Vancouver B fractures. These fractures are further classified into B1 if the femoral stem remains well fixed, B2 if the femoral stem is loose with good remaining bone stock, and B3 if the stem is loose with poor bone stock [7-8]. Historically, the recommended treatment for B1 fractures has been open reduction and internal fixation (ORIF), whereas the recommended treatment for a B2 fracture has been revision THA in addition to ORIF of the fracture [9-10].

The optimal treatment for Vancouver B fractures has been called into question by multiple studies, with ORIF alone demonstrating similar outcomes compared to revision THA for Vancouver B2 fractures [11-14]. Recent systematic reviews have now found similar reoperation rates for Vancouver B2 fractures treated with arthroplasty and ORIF [15]. Given similar no clear advantage to the outcome based on treatment selection alone, surgical training is likely to play a significant role in developing the treatment strategies for these complex fractures. The primary purpose of this study was to compare the outcomes of ORIF versus arthroplasty for Vancouver B2 fractures (OTA 32A1 [IVB2]) while evaluating the surgical training bias of the treating surgeon [16].

## Materials and methods

Forty patients treated with Vancouver B periprosthetic proximal femur fractures from 2007 to 2017 at a single institution were retrospectively identified through queries of the electronic medical record after institutional review board (IRB) approval. Demographic data, including age, gender, body mass index (BMI), and Charlson Comorbidity Index (CCI), was collected. Radiographic Vancouver classification was independently performed by both an arthroplasty and a trauma-trained surgeon using preoperative injury radiographs. Classification, mode of treatment, and treating surgeon specialty were analyzed. Nine were classified as B1 fractures, and 31 were classified as B2 fractures. Nine B2 fractures were confirmed by the review of the operative report stating the stem was loose. The remaining 22 B2 fractures were identified by agreement between the arthroplasty and trauma-trained surgeon upon review of the radiographs. Of the 31 patients with B2 fractures, 15 underwent revision arthroplasty and 16 were treated with ORIF. All patients underwent a re-operation of their THA with either a hip revision arthroplasty or open reduction and internal fixation of their hip. Nine patients received a cemented stem, and six patients received an uncemented stem. The type of procedure, revision arthroplasty, or ORIF was decided at the discretion of the treating surgeon.

Outcomes included mortality, revision, and reoperation for any reason, infection, and estimated blood loss. Fisher’s exact tests were used to evaluate associations between treatment modality and categorical variables. Wilcoxon rank-sum test was used to evaluate associations between treatment modalities and continuous variables.

## Results

There were no statistically significant differences in revision, re-operation, or infection between arthroplasty and ORIF at an average follow-up of 65 weeks. Additionally, there were no demographic differences between the groups with regard to age, gender, body mass index (BMI), or CCI (Table 1). There was one mortality in the arthroplasty group and five in the ORIF group (*P *= 0.18). There were two further re-operations in the arthroplasty group, each patient underwent irrigation and debridement followed by a revision arthroplasty, and one re-operation for aseptic femoral loosening three years following fixation in the ORIF group in which this patient underwent a revision arthroplasty. Median estimated blood loss was 700 cc in the revision arthroplasty group compared to 400 cc in the ORIF group (*P *= 0.04), but the median number of units transfused was statistically similar between revision arthroplasty (1 unit) and ORIF (0 units; *P* = 0.25; Table 2).

**Table 1:** Demographics of Vancouver B2 femur fractures. BMI, body mass index; ORIF, open reduction internal fixation

Variable (Median)	Surgical procedure		P
	Arthroplasty	ORIF	
Patients, *n *(%)	15 (48.39)	16 (51.61)	
Age (years)	79	77.5	0.97
Male, *n* (%)	7 (46.67)	8 (50)	0.99
BMI	28.28	25.53	0.23
Charlson Comorbidity Index	4	4.5	0.26

**Table 2:** Comparison of surgical metrics and outcomes by repair procedure among Vancouver B2 femur fractures. *Significance at alpha = 0.05. pRBCs, packed red blood cells

Variable (*n*)	Surgical procedure		P
	Arthroplasty, *n* (%)	ORIF, *n* (%)	
Patients	15 (48.39)	16 (51.61)	
Trauma training (20)	5 (33.33)	15 (93.75)	<0.01^*^
Arthroplasty training (11)	10 (66.67)	1 (6.25)	<0.01^*^
One-year mortality (31)	1 (7.14)	5 (31.25)	0.18
Infection (31)	3 (20)	2 (12.50)	0.65
Reoperation (31)	2 (13.33)	1 (6.25)	0.60
Revision (31)	2 (13.33)	1 (6.25)	0.60
Median estimated blood loss (mL or cc) (28)	700	400	0.04*
Median pRBCs transfused (25)	1	0	0.25

Cases treated by surgeons with fellowship training in arthroplasty were more likely to be treated with revision arthroplasty (10/11, 90.9%) than those treated by surgeons with fellowship training in trauma (5/15, 33.3%; *P *< 0.01).

## Discussion

Vancouver B2 fractures present a challenging problem to orthopedic surgeons. Recent evidence has demonstrated a trend different from the historical dogma of universally treating B2 fractures with revision arthroplasty, with research including systematic reviews showing similar results with revision arthroplasty and ORIF [11-13,15]. This study demonstrates that surgical decision-making is heavily influenced by the treating surgeons’ previous training. When Vancouver B2 fractures were treated by a trauma fellowship-trained surgeon, 93.75% of those patients underwent ORIF as their definitive treatment despite the historical teaching that these fractures require revision arthroplasty. However, despite this bias, there was no statistically significant difference in outcomes between the two treatment strategies. However, the arthroplasty group had greater estimated blood loss.

Mortality after surgical treatment of periprosthetic femur fractures is similar to that of native hip fractures [6]. However, it is unclear if treatment strategy can modify this outcome. Bhattacharyya et al. described a higher mortality rate in B2 fractures treated with ORIF citing weight-bearing status following treatment as a possible contributing factor [17]. In contrast, Gitajn et al. retrospectively reviewed B2 fractures treated with ORIF versus revision arthroplasty and found no difference in mortality concerning the treatment method [18]. At the authors’ institution, it is common to allow full weight-bearing following ORIF of periprosthetic femur fractures. Although there was no statistically significant difference, the trend toward increased mortality in the ORIF group (5) over the revision group (1) does indicate this as an outcome to further evaluate in future studies. Especially because the ORIF group was associated with lower blood loss, and if a bias was present, those treated with ORIF would likely be less severe or displaced fractures.

Limitations to this study include its retrospective nature, limited study size, and relatively short follow-up. Further, there was no characterization of the specific fracture patterns or prosthetic stem types. Minimally displaced fractures or more extensively coated stems would theoretically be more amenable to ORIF. It was not randomized, and treatment was influenced by the training of the treating surgeon. However, treatment *bias* may also be influenced by who the patient was directed to for treatment based on patient and injury characteristics that are difficult to capture. Radiographic loosening is subjective, and there is a spectrum of findings that may be present. Such fractures are often treated by the on-call surgeon at this institution, which depends on the day of the week, who is on call, and the complexity of the case. For example, if a stem is grossly loose radiographically, it is probably more likely to be referred to an arthroplasty surgeon from the outset. The converse is likely true with stems that may have shown only subtle signs of loosening. Further, the true stability of the stem was not confirmed in the majority of cases and had to be inferred based on a radiographic classification. Of the nine stems that were confirmed loose in the operative report, all were treated with revision. However, in every instance in which the operative report noted a loose stem, this was treated by an arthroplasty surgeon. Additionally, there was one case of revision for aseptic femoral loosening in the ORIF group, indicating stem ingrowth does not always occur with fixation. Finally, the outcome measures of revision, mortality, infection, and blood loss do not capture patient function or satisfaction, given the retrospective nature of the study.

## Conclusions

Despite the limitations, this study demonstrates similar results of ORIF and revision arthroplasty with Vancouver B2 fractures. While revision arthroplasty is still considered the gold standard, based on this study and others, similar short- and midterm outcomes can be achieved with ORIF alone in select Vancouver B2 fractures. However, it is important to note that no randomized controlled trials compare the two options, and this study and other published results are small series. The optimal treatment for the patient may also depend upon the option that the treating surgeon is most comfortable with, the characteristics of the fracture, the prosthetic stem in place, and the demographic or medical characteristics of the patient. Given the lower blood loss, ORIF may be attractive in patients at particular risk of cardiovascular collapse. Further research is necessary, with close attention to selection bias, to better understand the relationship between surgical treatment and mortality. 
